# Genomic variation reveals demographic history and biological adaptation of the ancient relictual, lotus (*Nelumbo* Adans.)

**DOI:** 10.1093/hr/uhac029

**Published:** 2022-02-19

**Authors:** Xingwen Zheng, Tao Wang, Teng Cheng, Lingling Zhao, Xingfei Zheng, Fenglin Zhu, Chen Dong, Jinxing Xu, Keqiang Xie, Zhongli Hu, Liangbo Yang, Ying Diao

**Affiliations:** 1 State Key Laboratory of Hybrid Rice, Lotus Engineering Research Center of Hubei Province, College of Life Sciences, Wuhan University, Wuhan 430072, China; 2 Guangchang White Lotus Research Institute, Guangchang 344900, China; 3 College of Biological Engineering, Henan University of Technology, Zhengzhou, Henan 450001, China

## Abstract

Lotus (*Nelumbo* Adans.), a relict plant, is testimony to long-term sustained ecological success, but the underlying genetic changes related to its survival strategy remain unclear. Here, we assembled the high-quality lotus genome, investigated genome variation of lotus mutation accumulation (MA) lines and reconstructed the demographic history of wild Asian lotus. We identified and validated 43 base substitutions fixed in MA lines, implying a spontaneous mutation rate of 1.4 × 10^−9^ bases/generation in lotus shoot stem cells. The past history of the lotus revealed that the ancestors of the lotus in eastern and southern Asia could be traced back to ~20 million years ago and twice experienced significant bottlenecks and population splits. We further identified selected genes among three lotus groups in different habitats, suggesting that 453 differed genes between the tropical and temperate group and 410 differed genes between two subgroups from Northeastern China and the Yangtze River–Yellow River Basin might play important roles in natural selection in the lotus’s adaptation and resilience. Our findings not only improve understanding of the evolutionary history of the lotus and the genetic basis of its survival advantages, but also provide valuable data for addressing various questions in evolution and protection for relict plants.

## Introduction

Relict plants belong to ancient plant groups that have witnessed Earth’s biological evolution. Relict plants have a long history and were widely distributed in the Cenozoic Tertiary period or earlier. Due to changes in geology and climate, they only exist in a small area, but retain the original morphology of their ancient ancestors [1]. Most related groups of relict plants are extinct and they are relatively isolated and evolve slowly [2].

*Nelumbo* is the sole genus in Nelumbonaceae, which belongs to Proteales, a group that originated in the Cretaceous ~140 million years ago (Mya) [3]. Compared with other relict plants, like *Metasequoia glyptostroboides*, *Ginkgo biloba*, *Liriodendron chinense*, and *Sequoia sempervirens* [4], which are confined to relatively narrow, discontinuous ranges in the world, lotus (*Nelumbo*) is widely distributed in Asia, Oceania, and America [5]. In addition, lotus also has important value in agriculture, religion, literature, art, and other cultural fields. In China, it has been domesticated for >7000 years and now there are >2000 lotus cultivars worldwide [6]. Owing to its rapid clonal growth and reproduction, lotus can quickly occupy growing space in shallow and slow-moving water. In Connecticut, USA, local government even treats lotus as an invasive plant [7]. In contrast to the plant’s vigorous growth, the lotus genome has evolved slowly and maintained low heterozygosity [8, 9]. In the past 5 years, many scientific studies have been conducted on the genome of lotus. Using high-resolution genetic maps and BioNano genome mapping, Gui *et al*. [10] improved the lotus genome and found ancient chromosome rearrangements. Zhao *et al*. [11] performed whole-genome resequencing and identified promising functional markers and candidates for molecular breeding. Fu *et al*. [12] studied the genetic diversity of the wild Asian lotus from Northern China and provided essential resources for the Chinese wild lotus. Recently, research on DNA methylation in the lotus genome has been greatly promoted and conducted successfully [13, 14]. However, studies on the mutation rate and demographic history of lotus are relatively few and evolutionary questions related to extinction, competition, and adaptation in lotus remain unclear.

Here, we report a chromosome-level *de novo* assembly of the lotus genome and performed somatic cell mutation analysis to identify the generation, maintenance, and distribution of genetic variations in lotus. Furthermore, we also inferred the demographic history of wild Asian lotus and identified genomic regions potentially under selection in ecological adaptation. Overall, our study not only provides an insight into the genetic structure and evolutionary history of relict plants, but also provides a theoretical basis for studying adaptive evolution and the increasing significance of asexual and sexual reproduction in plants.

## Results

### Sequencing and assembly of the lotus genome

Based on ~57.9-Gb Nanopore sequencing data of an Asian cultivated lotus, ‘Taikonglian No. 3’, we obtained a high-quality lotus genome assembly with contig N50 of 5.1 Mb and total genome size of 807.01 Mb (Supplementary Data Table S2). This assembly is ~10.5 times the length of the previously reported chromosome-level genome of sacred lotus ‘China Antique’ [14] (contig N50 484.3 kb). Further, BUSCO (Benchmarking Universal Single-Copy Orthologs) analysis showed that, of the 1614 expected embryophytic genes (embryophyta_odb10), 1521 (94.24%) complete BUSCO genes could be aligned to this lotus genome assembly (Supplementary Data Table S3), suggesting its high completeness and reliability. We also used the long terminal repeat (LTR) Assembly Index (LAI) to assess the quality of this assembly; the LAI value is 10.26 (>10), which indicates this assembly reaches reference level.

Further, a Hi-C library was constructed and sequenced to help cluster, order, and orient these contigs into chromosome-level scaffolds (pseudomolecules). A total of 974 369 806 clean reads (q30 > 93.69%) were obtained by Hi-C library sequencing. Of these reads, 260 022 704 were unique map reads, accounting for 53.37% of whole sequencing data, and 139 573 647 were valid interaction pairs, accounting for 53.68% of unique map reads. We used the 3D-DNA method and obtained a total of 1535 scaffolds (805 Mb, 99.82%), of which 553 (783 Mb, 97.1%) could be anchored to eight pseudochromosomes, leaving ~22 Mb of the scaffolds unplaced (Supplementary Data Table S4). The Hi-C genome interaction heat map (Supplementary Data Fig. S2) showed that eight chromosome clusters could be distinguished clearly and the interaction intensity of the diagonal position in each group was higher than that in the non-diagonal position with no obvious noise (strong interaction intensity) outside the diagonal line.

**Figure 1:**
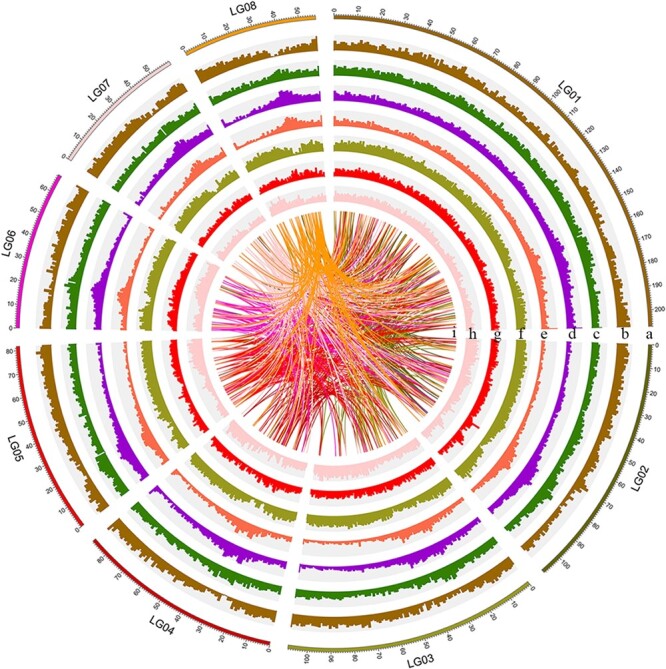
The landscape of the lotus genome. (a) Size (Mb) of the genome for each chromosome. (b) Density of genes [log_2_ (number of genes)]. (c) GC content (45.0–48.0%). (d) Density of TEs. (e) Density of retrotransposons. (f) Density of LTR retrotransposons. (g) Density of SNPs among the wild accessions. (h) Density of indels among the wild accessions. (i) Syntenic blocks. The interval size is 1 Mb.

In this work, we obtained a high-quality chromosome-level genome (Fig. 1), which we used as the foundation of a study of the demographic history and biological adaptation of *Nelumbo*.

### Repeat sequence identification and gene annotation

The newly assembled genome contains about 509.29 Mb (~63.11%) of repetitive sequences (Fig. 1c, Supplementary Data Table S5), which is more than that of *Arabidopsis thaliana* (~14%) and rice. Of these repeat sequences, transposable elements (TEs) amount to 484.55 Mb, accounting for 60.04% of the genome. LTRs in TEs amount to 219.64 Mb, representing 27.22% of the genome, and retrotransposons in LTRs amount to 38.05 Mb, representing 4.71% of the genome.

Subsequently, based on a repeat-masked genome sequence, we used a classic pipeline to predict protein-coding genes in the lotus genome and obtained a total of 28 274 genes (Fig. 1d; Supplementary Data Table S6), which was roughly the same as the number of protein-coding genes in *A. thaliana* (~30 000) but less than the number in rice (~40 000). Of these genes, 27 627 (97.71%) are located on chromosomes with an average density of 34 genes per megabase. The average number of exons per gene is 5.3 and the average exon length is 226.1 bp.

### Analysis of somatic mutations

#### Determination of single-nucleotide polymorphisms in shoot stem cells

Based on the principles of determination of homozygous or heterozygous sites, ~180.0 Mb sites in the lotus genome were filtered. Of the remaining 627.0 Mb sites, the number of heterozygous and homozygous sites was 2.6 and 624.4 Mb, respectively. Subsequent mutation analysis was based on these sites (Supplementary Data Table S4). Lotus mutation accumulation (MA) lines L1–L8 were sequenced using Illumina with the sequencing depth of 40× (Supplementary Data Table S7). Filtered data for each line were compared with the reference genome using the Burrows–Wheeler Aligner (BWA-MEM). We selected 758 SNPs (coverage of 20×, homozygosity 85%, and heterozygosity 30%) for further validation by Sanger sequencing. Of these, 506 sites were sequenced successfully and 45 SNPs were confirmed, and the positive rate was 45/506 = 8.89%. According to this positive rate, 22 true SNPs might be present in 252 predicted sites that failed to be sequenced, which is in the same order of magnitude as the number of mutations verified, and will not introduce bias to subsequent analysis. Based on reported empirical values, such as that in *A. thaliana* [15] and *Drosophila* [16], very low false negative (FN) rates (<2%) were found. Therefore, the maximum FN rate of 2% was used to estimate FN SNPs: 0.02 = FN/(FN + TP), where TP represents estimated true SNPs. When TP equals 45, the FN is 0.92, suggesting that we have only missed one SNP.

Stem cells in the shoot apical meristem are not single cells, but stem cell groups [17]. Mutations can be derived from vertex stem cells and peripheral stem cells. Mutations in vertex stem cells can be fixed, while in peripheral stem cells they may be fixed in lower branches [18]. The fixed mutations in stem cells can be kept in clones and transferred to the offspring, which is more important than the situation with other somatic mutations in the clone population. Although not much is known about the role of stem cells in lotus morphogenesis, it is certain that a mutation detected in both the leaf of the clone and its offspring is a mutation already fixed in stem cells of that clonal population. According to this principle, 43 of 45 SNPs were confirmed in the offspring of the corresponding MA line. That is, there are 43 SNPs mutations fixed among the MA lines (Table 1; Supplementary Data Table S8).

**Table 1:** Summary of fixed somatic mutations (base mutations) validated by Sanger sequencing.

MA line	Intergenic	Intron	CDS	Total	Mutation rate (×10^−9^) (bases/generation)	Standard error (×10^−9^)
L1	3	0	0	3	0.8	0.5
L2	5	0	0	5	1.3	0.6
L3	6	0	1	7	1.8	0.7
L4	6	1	0	7	1.8	0.7
L5	2	1	0	3	0.8	0.5
L6	4	0	0	4	1	0.5
L7	5	0	0	5	1.3	0.6
L8	7	2	0	9	2.3	0.8
All	38	4	1	43	1.4	0.2

#### Estimation of mutation rate

**Figure 2:**
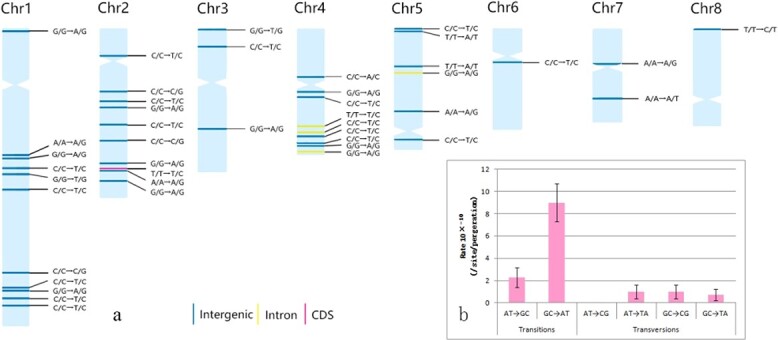
Distribution and mutation rate of fixed mutations. **a** Distribution of mutations across chromosomes. Labels indicate the type of mutation and colors their functional context. **b** Mutation rates of different mutation types. Error bars indicate standard errors of the mean.

We found that mutation accumulation lasted six generations (years) and detected 43 fixed mutations. The number of mutations that accumulated in each clone line was between three and nine, with no significant difference in the number of SNPs among individuals (Table 1). By combining the sites at which mutation was detected (~627 Mb) with the number of generations for which mutations accumulated (six), the mutation rate can be estimated. The calculated mutation rate of stem cells in each MA line was 0.8 × 10^−9^ to 2.3 × 10^−9^ bases/generation, and the average mutation rate was 1.4 × 10^−9^ bases/generation, which was slightly lower than that of annual plants, such as *A. thaliana* (7 × 10^−9^) [15] and maize (2.17 × 10^−8^ to 3.87 × 10^−8^) [19] (Fig. 2).

For ‘Taikonglian No. 3,’ an average of 15 nodes on the ortet produce a ramet, indicating that the terminal buds of ramets have undergone 15 budding processes. Each bud of the lotus can develop into a reproductive unit (with roots, stems, leaves, flowers, and axillary buds). Therefore, the estimated mutation rate of each reproductive unit of stem cells was 5.1 × 10^−11^ to 15.3 × 10^−11^ bases/phytomer/generation (average 9.3 × 10^−11^ bases/phytomer/generation). The above-ground part of the lotus is annual. From the perspective of the reproduction unit level, the spontaneous mutation rate of lotus stem cells is much lower than that of annual plants, such as *A. thaliana* and maize, but is equivalent to the somatic mutation rate of the giant duckweed, an aquatic clonal plant (7.92 × 10^−11^ to 2.38 × 10^−10^) [20].

In herbaceous plants, such as *A. thaliana* and tomato, axillary meristem cells are separated from apical meristem cells by seven to nine cell divisions [18]. If this is the general pattern of herbaceous plants, the average mutation rate in lotus stem cell division will not be higher than 1.0 × 10^−11^ to 1.3 × 10^−11^ bases/cell division, which was significantly lower than the average stem cell mutation rate of some species, for instance, *A. thaliana* (0.16 × 10^−9^ bases/cell division), *Escherichia coli* (0.26 × 10^−9^ bases/cell division) and *Saccharomyces cerevisiae* (0.33 × 10^−9^ bases/cell division) [21].

### Characteristics of single-nucleotide polymorphisms

Spontaneous mutations are unevenly distributed on chromosomes in plants such as *A. thaliana* [15], maize [19], and oak [22], as well as in lotus. The SNPs were found on each chromosome (Fig. 2a; Supplementary Data Table S9). The mutation rate of each chromosome was between 3.0 × 10^−9^ and 20.8 × 10^−9^ bases/generation. The frequency of mutations had a weak correlation with chromosome length. Mutations were relatively concentrated on the long arms of chromosomes 1, 2, and 4; mutations near telomeres were mainly distributed at the short-arm end, and few mutations were near to the centromere. Of the 43 SNPs, 38 occurred in the intergenic region, 4 in introns and 1 in the coding sequence (CDS) region (Supplementary Data Table S9). This mutation in the CDS region can change Thr to Ala at position 305 of the protein, encoded by a vacuolar cation/proton exchanger 3-like gene in lotus. Some studies have found that the mutation rate of heterozygous sites is higher than that of homozygous sites [23]. However, all mutations detected in lotus are homozygous to heterozygous. The highly homozygous genome of lotus [8] may be caused by long-term purification selection, but it seems that the lotus genome has been trying to increase heterozygosity during evolution and provide a basis for balanced selection [24]. Among the mutations, ~65% were of the G:C → A:T type (Fig. 2b), but the A:T → C:G type was not found, suggesting that the lotus genome is possibly evolving toward increasing AT content.

### Population structure and evolution analysis

#### Population structure analysis

To investigate the population structure and admixture of wild lotus, we downloaded whole-genome resequencing data of 32 wild lotus accessions from public databases and mapped them to the reference genome assembled in this study. After filtering, a total of 4 185 743 SNPs was detected across 32 wild lotus individuals, with an average of 5187 SNPs per megabase. According to their geographical origins, we divided wild Asian lotus accessions into three populations: SEA (Southeast Asia), YZYR (Yangtze River–Yellow River Basin), and NEC (Northeastern China). Besides, they can be also divided into two groups, corresponding to two ecological types, namely tropical (SEA) and temperate (NEC and YZYR).

**Figure 3:**
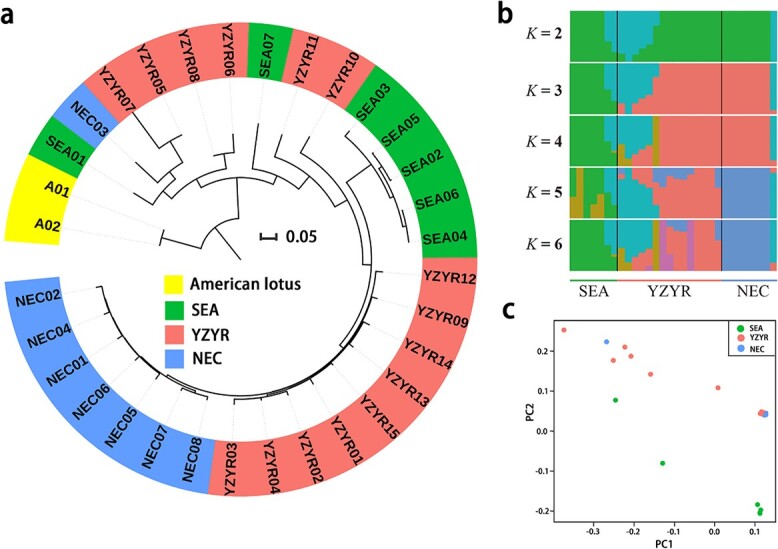
Phylogenetic relationships and population structure of wild lotus. **a** NJ phylogenetic tree of wild lotus samples using whole-genome SNP data based on genetic distances. **b** Population structure of wild lotus individuals based on ADMIXTURE analysis with *K* = 2–6. **c** PCA of wild Asian lotus.

Using American lotus as outgroup, based on genetic distance, we constructed the neighbor-joining (NJ) phylogenetic tree of 32 wild lotus accessions (Fig. 3a). The NJ tree showed that accessions that had the smallest genetic distance to American lotus were SEA01 (from Thailand), YZYR05 (from Jiangxi Province, China), and YZYR06 and YZYR08 (both from Yunnan Province, China), suggesting that current wild lotus populations in East Asia and Southeast Asia might originate from these three regions. In addition, the genetic distance of lotus accessions in Southeast Asia and China was quite large, while the genetic distance of two populations in China was relatively small, which confirmed that the difference between tropical and temperate lotuses was greater than that between two temperate lotuses. Among temperate lotuses, accessions from Northeastern China were closely related to those from the Yangtze and Yellow River Basins. Different dimensions correspond to different climatic conditions. The genetic difference between wild lotuses in China is consistent with their geographical distribution. Recently, Liu *et al*. [25] reported that lotuses in Northeastern China and lotuses in Southeast Asia had closer genetic relationship than those in the Yangtze River Basin, which was different from ours. We believe that our result is more reliable because of the greater sequencing depth and the higher quality of the reference genome we used, but it also depends on sample size and representativeness.

**Figure 4:**
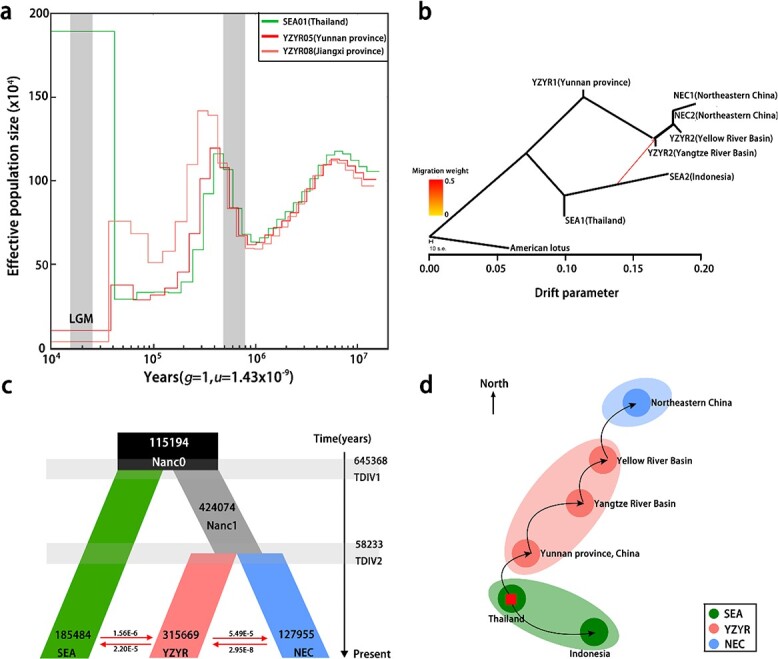
Demographic history and estimated migration routes of wild lotus in Asia. **a** Population size through time plot for the three wild lotus individuals with longest traceable history (SEA01, Thailand; YZYR05, Yunnan province; YZYR08, Jiangxi province) based on pairwise sequentially Markovian coalescent modeling. **b** TreeMix analysis of wild Asian lotus. **c** Schematic of demographic scenarios modeled using FASTSIMCOAL2. The number in each segment represents the estimated effective population size *N*_e_ and the red arrow indicates the estimated gene flow between lineages. The numbers on the vertical axis indicate the estimated divergence time of wild lotus populations. **d** Estimated migration routes of wild lotus in Asia based on demographic history. The red square represents the original centers of Asian lotus and the arrow shows the direction of population migration in the past history.

ADMIXTURE analyses at *K* = 2–6 recapitulated these findings: when *K* = 3, the wild lotus in Southeast Asia was distinctly separated from that in China; and up to *K* = 5 the accessions from Northeastern China could be separated from other accessions (Fig. 3b). These observations suggested that the genetic difference between the two temperate populations in China was small and the corresponding genetic relationship was close, while the genetic difference between the lotus groups in China and those in Southeast Asia was large, which was consistent with the NJ tree. Notably, the genetic composition of some accessions seemed complex, indicating that there might be high gene flow in these accessions.

Principal component analysis (PCA) of wild lotus (Fig. 3c) revealed that tropical and temperate lotus accessions could be separated by PC2 and roughly formed two clusters. Tropical lotus accessions were distributed in a dispersed manner, indicating the high levels of genetic diversity among them. In contrast to tropical lotus accessions, those from Northeastern China distributed centrally. It is possible that natural selection in tropical regions is relatively varied and complex, while in Northeastern China it is simple. Besides, similar to the results of the NJ tree, two temperate lotus groups clustered closely, indicating a close genetic relationship between them.

### Demographic history reconstruction

Using the pairwise sequentially Markovian coalescent (PSMC) method, we inferred the historical changes of lotus at effective population size *N*_e_, where the mutation rate and generation time were set to 1.43 × 10^−9^ and 1 respectively. The results showed that the earliest wild lotus accessions that could be traced were estimated to start at ~20 Mya, including ‘Lianhu Lian’ (from Jiangxi Province, China), ‘Puzhehei Bailian’ (from Yunnan Province, China), and ‘Chachoengsao lotus’ (from Thailand) (Fig. 4a). The origin time is roughly the same as that of other living fossil plants—ginkgo [26] and *Cercidiphyllum japonicum*. The effective population size of these accessions reached the first peak at ~7 Mya, and then declined rapidly and reached the lowest level at ~1 Mya, which was reduced by ~50% compared with the previous peak. This may be related to the coming of the glacial period, during which the climate was not conducive to the survival of animals and plants [27–29]. After that, the population size expanded rapidly and reached the second peak at ~0.45 Mya, and then the population size contracted again and reached the lowest level at ~0.1 Mya. Two bottleneck periods of lotus were at ~1 and ~ 0.1 Mya, which was also detected in ginkgo [26]. In addition, the most recent effective population size started to expand at 0.05 Mya. However, the effective population size of Chachoengsao lotus (SEA01) expanded abnormally recently, which was possibly caused by the insufficient power of PSMC in inferring more recent demographic history.

Subsequently, using American lotus as outgroup, we used ancestry graphs implemented in TreeMix to analyze the admixture events and genetic relationships between the seven populations of wild lotus. Actually, according to the geographic origins of wild lotus accessions, we further divided the three populations (SEA, YZYR, and NEC) into seven populations, of which two were from Southeast Asia, three from the Yangtze River–Yellow River Basin, and two from Northeastern China. The TreeMix analysis (Fig. 4b) showed that the populations from Southeast Asia and China diverged at first, but there was still gene flow between the two populations from Southeast Asia and China. Among the Southeast Asia populations, Thailand lotus and Indonesia lotus diverged further. By contrast, among the populations in China, the population split in order of YZYR1(Yunnan Province), YZYR2 (Yangtze River), YZR3(Yellow River), NEC2 (Northeastern China), and NEC1 (Northeastern China).

Finally, we used Fastsimal2.6 to infer the past demographic histories of the three groups using the joint site frequency spectrum (SFS) calculated within the 4-fold degenerate synonymous site (4DTv) of the lotus genome. We tested a total of 16 different models and performed the log likelihood test and akaike information criterion comparison for all models to determine the best-supported model (Supplementary Data Fig. S3, Supplementary Data Table S11). Among the 16 models, model 3 and model 4 showed significantly higher support than the others. Considering the phylogenetic tree and the results of PSMC and TreeMix analysis, model 4 (Fig. 4c) was relatively more reasonable. This model showed that the wild lotuses of Southeast Asia and China diverged from their common ancestor at ~0.64 Mya and the divergence of two lotus populations from China occurred at ~0.06 Mya. The two divergence events of wild lotus populations occurred after the bottleneck period (after the lowest point in the PSMC diagram) (Fig. 4a), and the effective population size of the three populations was roughly consistent with that in the PSMC analysis. Estimates of bidirectional gene flow between the lotus populations in Southeast Asia and Yangtze River–Yellow River Basin were low (1.56 × 10^−6^/2.20 × 10^−5^), and the same was true for the two populations from the Yangtze River–Yellow River Basin and Northeastern China (5.49 × 10^−5^/2.45 × 10^−8^). Notably, estimates of gene flow from the lotus population in the Yangtze River–Yellow River Basin to two other lotus populations were significantly higher than those from the other two lotus populations to populations in the Yangtze River–Yellow River area, which may be caused by the larger effective population size of lotus in the Yangtze River–Yellow River Basin.

### Genetic changes underlying biological adaption

#### Genetic differentiation among lotus groups

**Figure 5:**
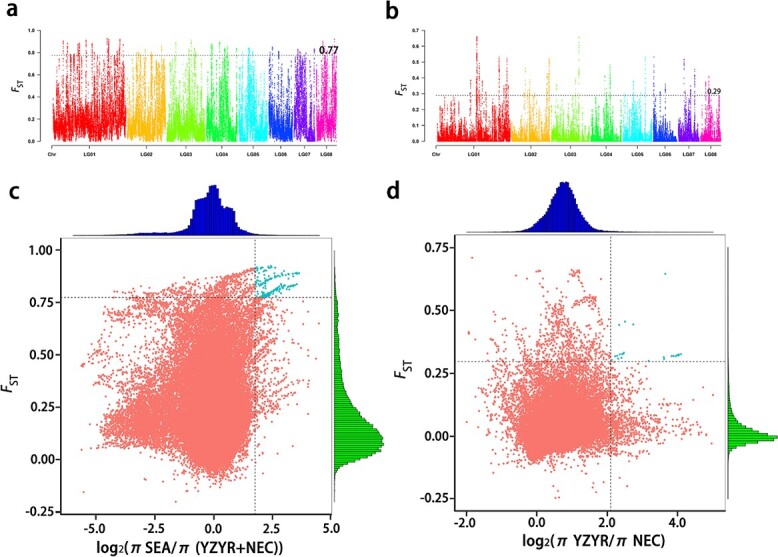
Selected sweep analysis of wild Asian lotus. **a***F*_ST_ values between tropical lotus and temperate lotus. **b***F*_ST_ values between two temperate lotus populations. The black line represents the top 99% threshold value. **c** Distribution of log_2_*π* ratios and *F*_ST_ values between tropical lotus and temperate lotus. **d** Distribution of log_2_*π* ratios and *F*_ST_ values between two temperate lotus populations. The candidate selective regions are shown as blue points.

Based on the genetic relationship and geographical distribution, the wild lotus was divided into tropical and temperate lotus groups, and the temperate lotus was further divided into two subgroups—the Yangtze River–Yellow River Basin subgroup and the Northeastern China subgroup. The *π* values of different groups were calculated at the whole-genome level with a window of 100 kb and a step of 10 kb (Fig. 5a). The results showed that *π* values for the tropical group, the Yangtze River–Yellow River Basin subgroup, and the Northeastern China subgroup were 1.0 × 10^−3^, 1.0 × 10^−3^, and 0.7 × 10^−3^, respectively. The *π* value of the Northeastern China subgroup was smaller than that in the Yangtze River–Yellow River Basin, implying that a bottleneck event possibly took place in the Northeastern China subgroup.

In addition, the *F*_ST_ value was calculated for two comparisons: tropical lotus versus temperate lotus and Yangtze River–Yellow River Basin lotus versus Northeastern China lotus (Fig. 5b). According to the *F*_ST_ values of the different groups, the genetic differentiation between tropical lotus and temperate lotus was more obvious than that between the two temperate lotus subgroups. Interestingly, a 10-Mb region in chromosome 1 (LG01) might be a candidate selection hot spot; when we used an *F*_ST_ value of 0.77 (99%) as the threshold to screen significant selected sites between the tropical and temperate lotus groups we found most of the selected sites located and clustered on these regions in LG01. Some genes in these regions, for instance *OF00315*, encoding an acyltransferase protein, whose homologous gene (*at5g2394*) in *A. thaliana* could alter cuticle layer, might play import roles in adaptation to different environments between tropical and temperate areas.

### Environmental adaptation and natural selection

The environmental factors in the growing areas of the three lotus groups were obviously different, which resulted in a different selection pressure for the corresponding lotus group. Combining the *F*_ST_ and *θπ* analyses, the top 5% sites with significant differences were identified as the selected region (Fig. 5c and d). Among these regions, there are 453 differed genes between tropical and temperate lotus (Supplementary Data Tables S12 and S13), and 410 differed genes between the two subgroups of temperate lotus (Supplementary Data Tables S14 and S15).

There is a big difference between temperate and tropical climates. The latitude of the temperate area is higher and the four seasons are distinct, whereas the tropical area is lower and is divided into dry and rainy seasons. The differences in environment led to obvious differences in morphology and growth characteristics between temperate lotus and tropical lotus. The biggest difference in morphology between them is whether the rhizome is enlarged. As expected, among the selected genes, several genes related to rhizome morphogenesis were also identified, such as expansin-like A1, *cyp714a* (*at5g24910.1*). Expansins play important roles in plant morphogenesis by expanding cell walls and elongating roots and stems [30], and the *cyp714a* gene is related to the increase in biomass and organ size [31]. Lotus in the tropics grows and blooms all year round, while lotus in the temperate zone grows and blooms in spring and summer, and goes into dormancy in autumn and winter. The differed genes between tropical lotus and temperate lotus were significantly enriched for the gene ontogeny (GO) term ‘recognition of pollen’ (go: 0048544). The main function of these genes is to identify and reject genetically similar pollen through the pistil to ensure outcrossing [32, 33]. Thus, compared with temperate lotus, the characteristic of continuous growth of tropical lotus makes it more prone to self-pollination. Long-term self-pollination and asexual reproduction lead to the decline of genetic diversity and increased extinction risk in the population [34, 35]. Different selection pressures on sexual reproduction between tropical lotus and temperate lotus lead to differences in pollen recognition-related genes. In addition, light conditions are also different between the temperate and tropical areas. Light intensity is greatest in the equatorial region, and decreases with increasing latitude. We found that several genes related to photosynthesis were also under selection pressure. For example, photosystem II oxygen evolving complex (*OF00512*/*OF15652*/*OF25237*) is involved in water splitting and oxygen release in photosynthesis [36, 37].

Compared with the Yangtze River–Yellow River Basin, climatic conditions in Northeastern China are not the most suitable growth environment for lotus. Notably, the annual average temperature in the three provinces is low, and the number of months of the year below 0°C average temperature can reach eight [38]. Low temperature is an important environmental selection factor. A cold-inducible gene encoding an active cationic peroxidase (*at1g05260.1*) was screened out, which was exclusively expressed in etiolated seedlings and roots of adult plants in *A. thaliana* [39]. Northeastern China is at a higher latitude and receives a long period of sunshine, which increases the possibility of DNA damage caused by UV in the plant. Some genes for DNA repair are strongly selected, such as NAD + ADP ribosyltranferase (*at5g22470.1*) [40], 8-oxoguanine-DNA glycosylase 1 (*at1g21710.1*) [41], and DNA polymerase epsilon subunit B2 (*at5g22110.1*) [42]. Moreover, poly ADP-ribose polymerase 3 (*at5g22470.1*) [43], mitochondrial hso702 (*at5g09590.1*) [44], and glutathione *S*-transferase F11 (*at3g03190*) [45] also play an important role in stress resistance. Black soil and chernozem are the main soil types in northeastern China, whereas brown, red, and cinnamon soils are dominant in the middle and lower reaches of the Yellow River and Yangtze River (http://geodata.pku.edu.cn), so that types and contents of metal ions in the soil and water are different. This difference may affect genes related to metal ion metabolism. For instance, the genes encoding vacuolar iron transporter 1 (*at2g01770*) [46] and a putative nodulin-like21 gene belonging to the vacuolar iron transporter (VIT) family [47] were found among the selected genes.

## Discussion

### Low mutation rate plays an important role in survival strategy of lotus

Although abundant variation can provide more choices for evolution, the low mutation rate of lotus in stem cells is conducive to maintaining the genetic stability of stem cells, which is of great significance for seed maintenance. Due to the limited propagation distance of clones, stable species make clonal progeny more adaptive to the living environment of the original place and reduce the pressure of environmental selection. By contrast, although the mutation rate in stem cells is low, accumulation of mutations in lotus stem cells is closely related to the division time (iterations). The more vigorous the clonal growth, the more stem cell mutations may accumulate, resulting in variation accumulation among ramets and thus increasing diversity within the clone population. Theoretically, according to the clonal growth model of lotus, if the average annual growth of the rhizome is 15 nodes, the number of iterations per plant will reach 215, or 32 768 ramets will be produced. In the clonal plant population, ramet density plays an important role in the evolution of plant life history [64]. However, according to field data, a single plant of ‘Taikonglian No. 3’ can produce ~200 ramets at most in 1 year. Therefore, it seems that variation within the clonal population of lotus is controlled at a low level.

During spontaneous mutations in plants, harmful mutations are produced, which affect plant survival. For example, most *A. thaliana* tissues are exposed to light, so most G:C → A:T transitions in *A. thaliana* are related to UV exposure [15]. From the growth mode and structure of lotus, the main part of lotus is under water. In particular, lotus buds are always underground, and the outer layer of buds is a thick sheath. The inner part of buds forms a nested structure, which closely surrounds the meristem. This effectively reduces the impact of the external environment, especially UV, on the meristem, and reduces the number of stem tip stem cell mutations caused by UV. In addition, lotus buds contain enzymes of the antioxidant system, namely Mn-superoxide dismutase (Mn-SOD), CuZn-superoxide dismutase (CuZn-SOD), ascorbate peroxidase (APX), and catalase (CAT). CuZnSOD is located in the nucleus and cytoplasm simultaneously, so that CuZnSOD in the nucleus can directly participate in the clearance of reactive oxygen species in the nucleus and protect nuclear DNA from oxidative damage [48]. Lotus shoot stem cells are well isolated from adverse environmental factors by the plant’s life style, morphological structure, and physiology, as far as possible, reducing the risk of DNA damage caused by the external environment, and reducing the number of resulting mutations, thus reducing the risk of harmful mutation.

In asexual propagation, the mutation rate of stem cells in the shoot of lotus is relatively low compared with other plants, which reduces the chance of selection. In sexual reproduction, the lotus fruit, with a hardy dry coat, makes germination in the natural state a challenge, which leads to slow generation replacement in the lotus. However, the mutations accumulated during growth can be maintained in the clonal population, and newly generated stem cell mutations can be spread in time with the aid of seeds, and can even be retained for up to 1000 years [49], which can be used for environmental selection for a long time in the future. Although this survival strategy leads to slow evolution, it may be the most competitive survival strategy of lotus.

### Migration routes of lotus

According to the genetic relationship and the demographic history analysis, the ancestors of lotus in Asia are possibly from Thailand and spread to high latitudes and low latitudes (Fig. 4d). At present, lotus is widely distributed in China. What we have always been interested in is how it survived the fourth Ice Age and migrated northward. Paleoclimatological research found that in the last glacial maximum in the fourth glacial period, the temperature in Northeast China dropped by 6–11°C, precipitation was 30–40% that of modern times, the temperature of North China and the middle and lower reaches of the Yangtze River dropped by 5–10°C, and precipitation was 30% in the middle and lower reaches of the Yangtze River [50, 51]. Lotus is a typical heat-loving aquatic plant, and the optimum temperature for its growth and development is from 25 to 30°C. According to the climatic characteristics of the Ice Age, the northeast and north regions of China are not suitable for lotus growth, while temperature and precipitation conditions in the middle and lower reaches of the Yangtze River and South China can support the survival of lotus. Therefore, the survival space of lotus might have been compressed to the south of China in the last glacial maximum. The two wild lotus accessions with the longest traced history in this study are from Erhai Lake in Yunnan Province and Lianhu Lake in Jiangxi Province. However, due to the barrier of the Yunnan–Guizhou Plateau, lotus in Erhai Lake and its adjacent areas could not migrate to Yangtze–Yellow River Basin. Lianhu Lake in Jiangxi Province belongs to the Poyang Lake area, the largest freshwater lake in China, belonging to the Yangtze River system. The nearby lotus of Lianhu Lake surviving in the glacial maximum is the most likely ancestor of lotuses existing in eastern and northern China. After the temperature rose in the interglacial period, the lotus migrated northward and spread all the way to the Yellow River Basin and then to northeastern China. The genetic distance of wild lotus populations is consistent with that of geographical distribution, which supports our proposed lotus migration route in China (Fig. 4d).

Our research shows that at the genomic level lotus plants in different growth environments have acquired mutations, resistance, and damage repair capabilities suitable for them to survive in the existing environment. Although lotus is more widely distributed than other relic plants, it is worrying that the population of wild lotuses is shrinking, which increases the possibility of species extinction. Human activities continue to encroach on the living space of other organisms, and a long-term decaying population may be more sensitive to the threat of human activities [52]. At present, searching for the wild lotus population in South China is a challenge, which also leads to the inability to determine the genetic relationship between wild lotus in South China and those in the middle and lower reaches of the Yangtze River. According to the climatic conditions of the fourth glacial period, the South China lotus might be one of the ancestors of the modern wild lotus in North China. However, from the geographical point of view, it is certain that if the lotus in South China migrates to the north, it needs to pass through the middle and lower reaches of the Yangtze River.

## Materials and methods

### Plant materials

The seed lotus cultivar ‘Taikonglian No. 3’ was kindly provided by the Guangchang White Lotus Research Institute and planted at the Lotus Research Center, Wuhan University. To construct the MA lines, one healthy rhizome only with the apical bud was planted in a pot (L0) in 2011 as the ancestor of the MA lines. In the spring of 2012, eight of the rhizomes propagated by L0 were carefully selected and planted individually as eight MA lines (L1–L8) (Supplementary Data Fig. S1). Each line was propagated by a single rhizome for 6 years (2012–17). In 2017, leaves of each MA line were collected for sequencing. Meanwhile, seeds of each flower in the MA lines were collected and kept separately. Among the MA lines, L3 was used for further genome assembly. ‘Taikong Lian No. 3’ is one of the most popular seed lotus cultivars that are widely planted in China. This cultivated lotus reference genome will play important roles in future molecular breeding and genetic improvement of the Chinese cultivated lotus.

### Data sources

Resequencing data on a total of 32 wild lotus accessions were downloaded from the National Center for Biotechnology Information (NCBI) (Supplementary Data Table S1).

### Genome sequencing, assembly and annotation

#### Illumina, Nanopore, and Hi-C sequencing

DNA was extracted from fresh leaves using the CTAB method [53]. Paired-end sequencing DNA libraries with ~500 bp inserted fragments were constructed and sequenced by the Illumina HiSeq 2500 sequencing platform. Then, using fastp [54] (v0.12.6), raw reads, including adaptor-containing reads, N-containing reads (>10%) and low-quality reads (alkali base number Q ≤ 5 was >50% of total reads) were removed.

The standard method [55] was used to sequence L3 of ‘Taikonglian No. 3’. First, high-quality DNA was extracted with the Qiagen^®^ Genomic kit, and the library was constructed. Then, the DNA was sequenced using Oxford Nanopore Technologies’ PromethION and GridION X5 sequencers to obtain original sequencing data.

The standard method [56] was used to sequence L3. First, chromatin with improved quality was extracted to construct the library and then sequenced using the Illumina NovaSeq sequencing platform to obtain 150 paired-end reads. Subsequently, the raw Hi-C reads were filtered with fastp [54] (v0.12.6) to obtain clean reads.

#### Genome assembly and Hi-C scaffolding

The Nanopore long reads were first corrected by Nextdenovo (v2.0-beta.1; https://github.com/Nextomics/NextDenovo) and then assembled into contigs with wtdbg (v1.2.8) [57]. The assembled genome sequence was then corrected using Pilon [58] based on Illumina short reads. We performed GC depth analysis on the assembly results and checked whether genome GC was abnormal and depth coverage was uniform to determine whether the sample was contaminated by other species and sequencing quality acceptable. The quality of the genome assembly was assessed using BUSCO (v 3.0.2) [59] and LTR LAI [60].

Then, all Hi-C reads were mapped to scaffolds generated in the previous step using bowtie2 (v2.3.2) [61]. Ligation sites in scaffolds were detected with HiC-Pro (v2.10.0) [62] by aligning 50 ends of reads back to scaffolds with the option –r hindiii. The number of valid interaction pairs was calculated, and valid interactions were used to build interaction matrices and draw a heat map with Juicebox (v1.8.8) [63] and HiC-Pro. Finally, the scaffolds were clustered, ordered, and oriented onto chromosome-level superscaffolds using LACHESIS (v2e27abb) [64].

#### Repetitive sequence identification and gene annotation

Simple sequence repeats (SSRs) and tandem repeats (TRs) were searched by GMATA (v2.2) [65] and Tandem Repeats Finder (v4.09) [66], respectively. MITE-Hunter (v2011) [67] was used to search miniature inverted-repeat transposable elements (MITEs) and create a MITE library file. LTR_Finder (v1.05) [68] and LTR_Harvest (v1.5.9) [69] were used to search for LTRs and then, using LTR_Retriever (v2.9.0) [70], results from both LTR searches were merged and an LTR repeat library file was constructed. The two libraries were integrated to form a TE library file (te.lib). RepeatModeler (v 2.0.1; http://www.repeatmasker.org/RepeatModeler/) was used to search for the repeat sequence and created a *de novo* library file (repmod.lib). With there being many unknown repeats in repmod.lib, we further used TEclass [71] to classify them. Finally, RepeatMasker (v 4.1.1; http://www.repeatmasker.org) was used to search known and novel TEs by mapping sequences against the te.lib, repmod.lib, and RepBase TE libraries (https://www.girinst.org/repbase).

Based on the repeat-masked lotus genome sequence, protein-coding gene prediction was performed using three strategies: *ab initio* prediction, homology-based prediction, and transcriptome-based prediction. For *ab initio* prediction, Augustus (v3.1) [72] was used to predict protein-coding genes based on a model trained by the classic pipeline. For homology-based prediction, GeMoMa (v1.3.1) [73] was used with the genome sequences and gene annotation files from *A. thaliana*, *Carica papaya*, *Vitis vinifera*, and *Brachypodium distachyon*. For transcriptome-based prediction, we extracted RNA from a mixture of tissues, including flower, leaf, rhizome, and root and then performed RNA-seq. Based on a total of ~19 Gb of transcriptome sequencing data, pasa (v2.0.2) [74] was used to predict gene structure. This RNA-seq data can be downloaded from NCBI with accession numbers SRR13674321–SRR13674323. Finally, all gene annotation results generated by the three methods above were evaluated by EVM (v1.1.1) [75] and TransposonPSI (v2013) (http://transposonpsi.sourceforge.net/).

### Somatic mutation analysis

#### Determination of homozygous or heterozygous sites

Genomes of clonal plants in nature are heterozygous. To identify spontaneous mutations in somatic cells, both homozygous and heterozygous mutations were investigated in this study. As reported [15, 19, 22, 76], coverage and base support are the most important parameters for the determination of homozygous sites. Therefore, we set the following principles for the determination of homozygous sites. (i) The coverage of candidate sites must be >20. (ii) The base of a site in a sample is inconsistent in the reads. If the number of reads of a base is ≥85%, it is considered a homozygous site and the base at this site is a true base. (iii) If there are two different bases at a certain site and the number of reads of a base is between 30 and 50%, the site is a heterozygous site and two different bases in the site are the true bases of the site. Although this filtering may lose some sites, it is beneficial for more accurate subsequent analysis.

#### SNP calling and validation

First, candidate SNPs were obtained by comparing resequencing data of L1–L8 to the filtered reference genome, and the number of reads supported by each site was obtained using SAMtools (v1.3.1) mpileup [77]. Then, candidate SNPs were screened using the consistency strategy. Among eight lines, seven had the same genotype, and one that was inconsistent with others. We selected SNPs using a filter of 85% homozygosity and 30% heterozygosity for Sanger sequencing.

#### Mutation transfer to offspring

During mutation transfer to seeds, the probability of a gamete carrying a mutation is 50%. The probability of no mutation in all fertilized eggs is (1/2)*^n^*, where *n* is the number of seeds. In our test, the number of seeds in each line was >10, and the probability of not detecting the transmissible mutation was less than (1/2)^10^ = 0.0009765625. Therefore, it is certain that transmissible mutations of the line can almost be detected effectively in the offspring. On this basis, all the SNPs identified from leaves were checked in the offspring of the corresponding lotus lines.

#### Estimation of mutation rate

Temperate lotus blooms once a year and 1 year can be regarded as a generation. Therefore, the mutation rate (*R*) of lotus shoot stem cells was calculated as follows: $R=\frac{\frac{m1}{n}}{m}$, where *m*1 is the number of SNPs in the sample, *n* is the age (generations) of accumulated mutations, and *m* is the number of detection sites. If each node of the lotus is a reproductive unit, the mutation rate of shoot stem cells in each reproductive unit is *R*/*K*, where *K* is the number of nodes where the lotus rhizome has experienced growth in 1 year (generation).

### Population structure and evolution analysis

#### Resequencing data processing, mapping, and variant detection

Genome resequencing data of 32 lotus accessions were downloaded from NCBI and after filtering by fastp (v0.12.6) [54], and were aligned to the reference genome using BWA (v0.7.13) [61] with default parameters. Then, SAMtools (v1.3.1) was used for sorting and converting them to the BAM format. PCR duplicates were marked using MarkDuplicates in the Genome Analysis Toolkit (GATK, v4.0.10.0; https://github.com/broadinstitute/gatk), and the index file was generated using SAMtools. HaplotypeCaller and GenotypeGVCFs in GATK were used to identify and extract SNPs and indels. Finally, raw SNPs were filtered with VarianFilteration in GATK with the parameters: —filter-expression ‘QD < 2.0 || MQ < 40.0 || FS > 60.0 || SOR > 3.0 || MQRankSum < -12.5 || ReadPosRankSum < −8.0’ —filter-name ‘SNP_filter’.

#### Genetic relationship and population structure analysis

The phylogenetic tree was constructed with the NJ method based on the whole-genome SNP data using MEGA (v7.0.14; https://www.megasoftware.net/) and the online website ITOL (https://itol.embl.de/) was used to assist visualization of the tree. Population structure and admixture analyses were performed using ADMIXTURE (v1.3.0; http://dalexander.github.io/admixture/download.html). PCA was performed using GCTA (v1.26.0) [78]. Images of population structure and PCA were drawn with R (v3.6.3; https://www.r-project.org/).

#### Reconstruction of demographic history

Firstly, we used the PSMC (v0.6.5) [79] method to infer the historical dynamics of effective population size over time and estimate divergence times of different lineages. The estimated mutation rate and generation time were set to 1.43 × 10^−9^ and 1, respectively. Then, we further used TreeMix (v1.13) [80] to infer population splits and mixtures of seven lineages, including SEA1, SEA2, YZYR1, YZYR2, YZYR3, NEC1, and NEC2. Finally, we used fastsimcoal (v2.6) [81] to simulate population history. A total of 16 divergence models in fastsimcoal were tested and model comparison was based on the maximum likelihood value. The model with the higher Akaike weight value was chosen as the optimal model.

### Biological adaptation analysis: identification, annotation and enrichment analysis of selected genes

The nucleotide diversity (*π*) for each population and *F*_ST_ between different subpopulations were calculated by VCFtools (v0.1.13) with the window size of 100 kb and the step size of 10 kb. Genomic regions of strong selection signals were determined by the top 5% *F*_ST_ and the top 5% log_2_*π*. Genes across these genomic regions were chosen as candidate selected genes. Then, using blastp (v2.10.1; − evaluate 1e-5, − Max_target_SEQS 1), protein sequences of selected genes were aligned to the KEGG (Kyoto Encyclopedia of Genes and Genomes) database (https://www.kegg.jp/). InterProScan (v5.36–75.0; https://github.com/ebi-pf-team/interproscan) was used to compare the gene protein sequence with the Pfam database with default parameters. The InterPro ID on the comparison was converted into its corresponding GO annotation information and classified. We performed GO and KEGG functional enrichment analysis based on the gene annotation information using ClusterProfiler (v3.14.3) [82], a gene enrichment analysis package in R.

## Acknowledgements

This work was partially supported by the Technology Innovation Project of Hubei Province of China (Major Program) (No. 2019ABA108), the Science and Technology Supporting Program of Jiangxi Province (20132BBF60018/20141BBF60014), the National Science and Technology Supporting Program (No. 2012BAD27B01), and Fundamental Research Funds for the Central Universities (2042015kf0244).

## Author contributions

X.Z., Z.H, L.Y., and Y.D. conceived and designed the study; Y.D., X.Z., T.W., T.C., L.Z., F.Z., X.F.Z., C.D., J.X., and K.X. performed the research; T.W., C.T., Y.D., and X.Z. analyzed the data; Y.D., X.Z., and Z.H. wrote the paper.

## Data availability

All raw genome sequencing data have been deposited under NCBI BioProject PRJNA698756 with accession numbers SRR13617441–SRR13617446. The genome assembly and gene annotation file can be downloaded from Comparative Genomics (CoGe) (https://genomevolution.org/CoGe/GenomeInfo.pl?gid=60468).

## Conflict of interest

The authors declare no competing interests.

## Supplementary Material

Web_Material_uhac029
