# Supplementary material for: Genomic variation reveals demographic history and biological adaptation of the ancient relictual, lotus (Nelumbo Adans.)
Source: Hortic Res. 2022 Feb 19;9:uhac029. doi: 10.1093/hr/uhac029 (PMC9039500; doi:10.1093/hr/uhac029)
Supplement: Web_Material_uhac029 [file web_material_uhac029.zip › SI_for_HR.docx]

**Genomic variation reveals demographic history and biological adaptation of the ancient relictual, lotus (*Nelumbo* Adans.)**

**Note:** All the supplementary tables are in the excel file (SI_tables.xlsx). With some supplementary tables are so big that they are hard to be seen clearly and understood easily if we show them in WORD file, we keep them in the EXCEL file.

Fig. S1. Propagating scheme of the individuals for the mutation accumulation


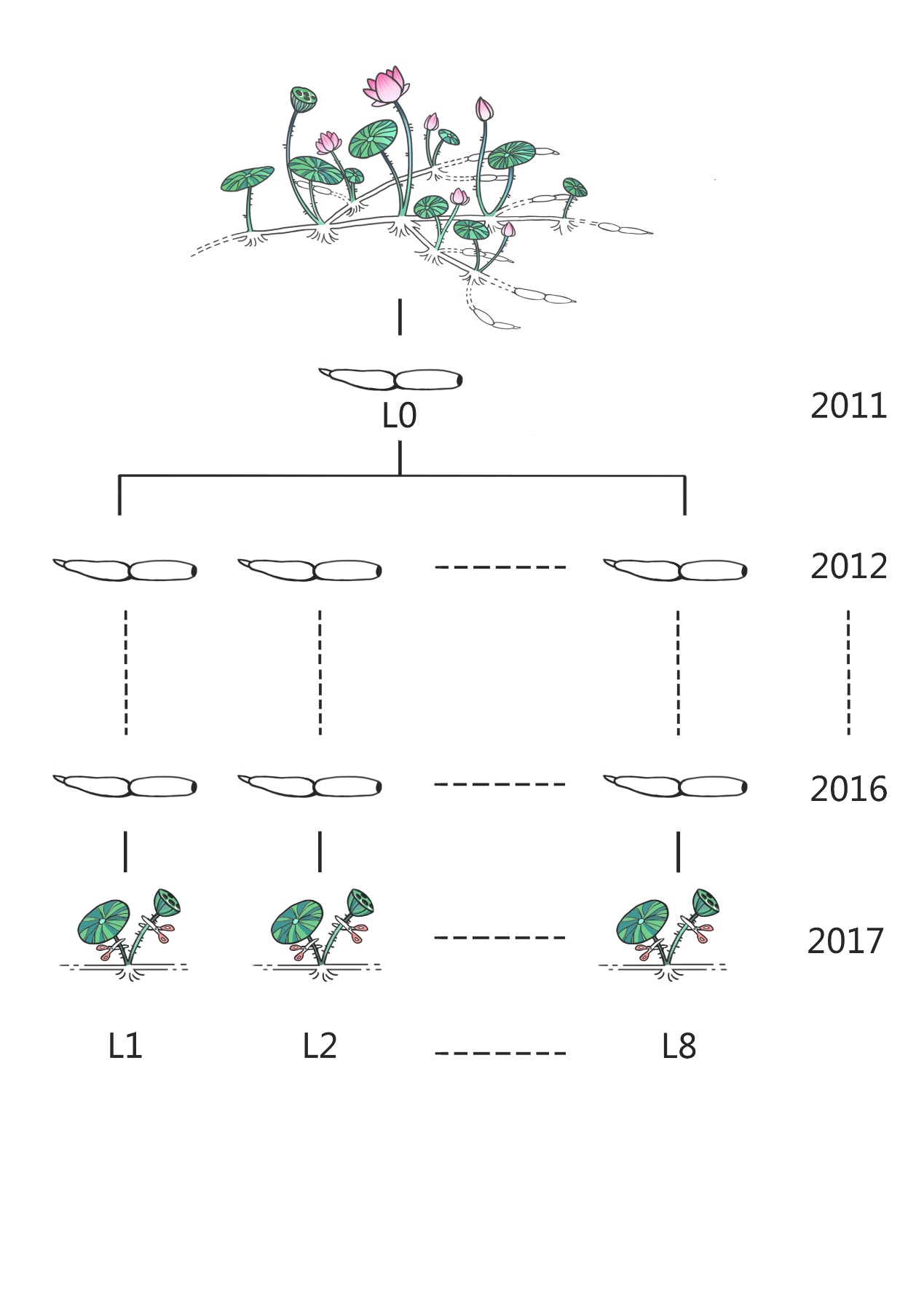


Fig. S2. Figure S2 Heatmap showing Hi-C interactions at 100-kb resolution. Intrachromosomal interactions displayed as anti-diagonal patterns.


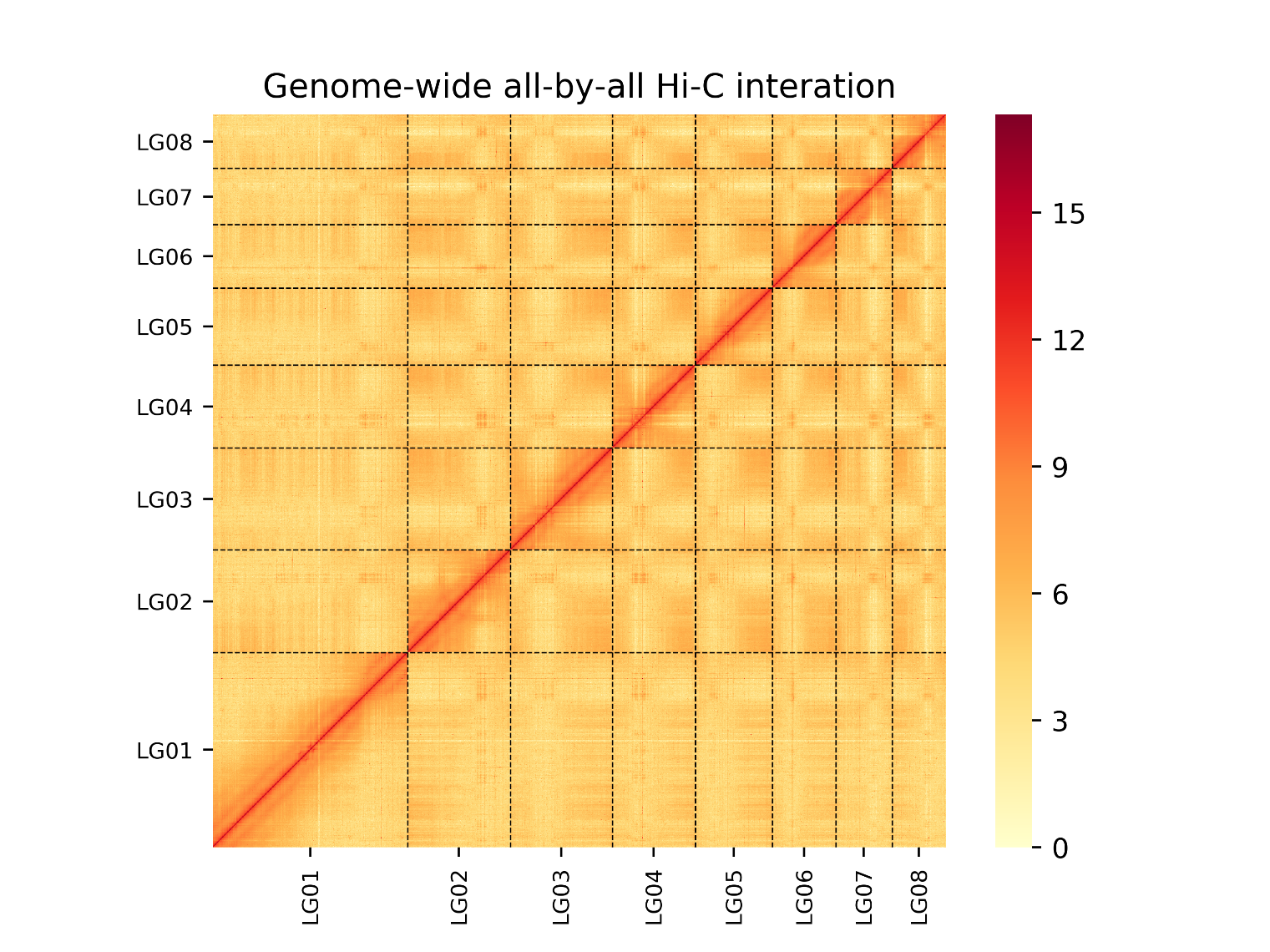


Fig. S3. 16 models tested in fastsimal2.6


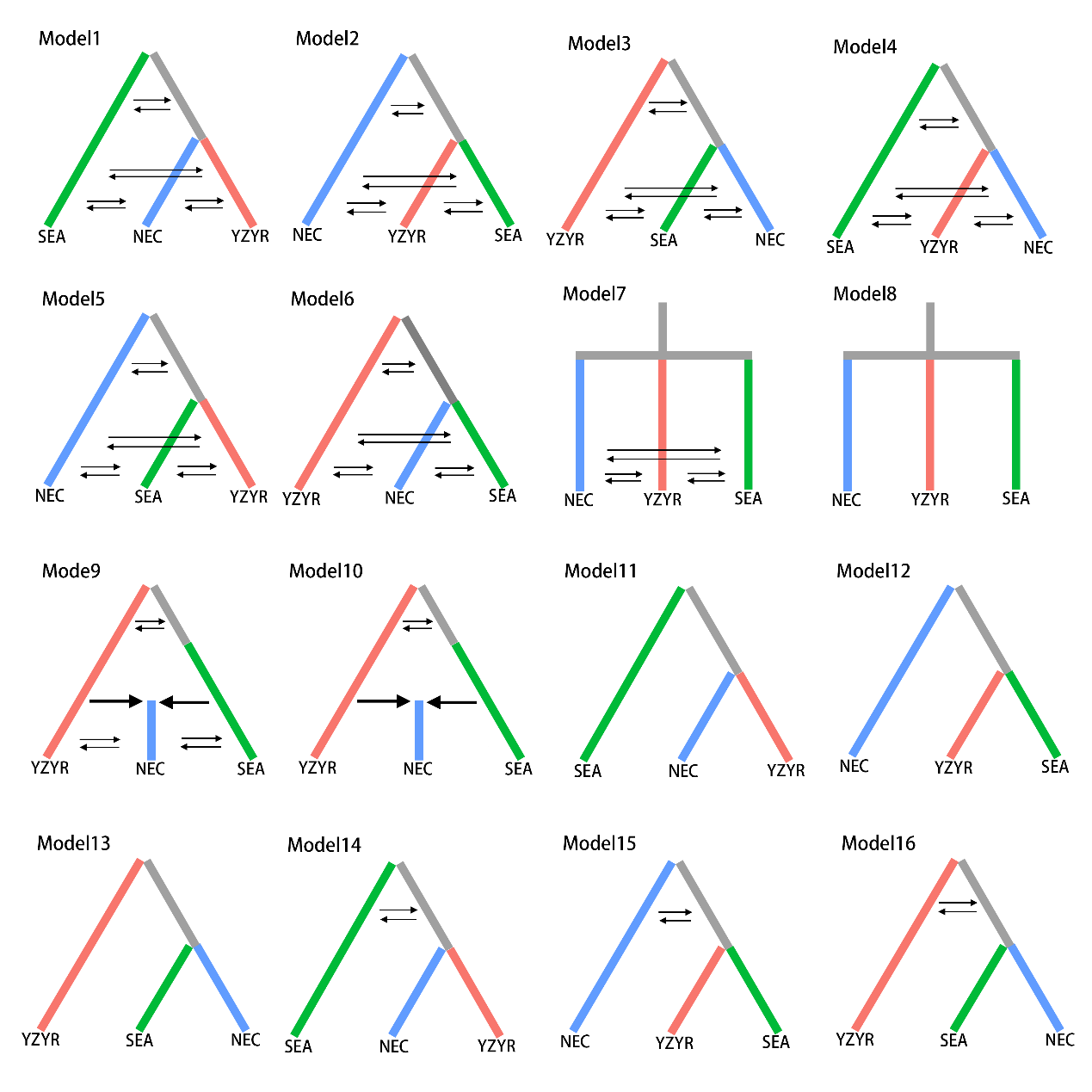


Table S1. Summary of wild lotus accessions

Table S2. Summary of Nanopore sequencing

Table S3. Result of BUSCO-prediction

Table S4. Pseudomolecules of lotus reference genome "RL3"

Table S5. Summary of repeat sequences in RL3

Table S6. Summary of gene annotation

Table S7. Sequencing result of the mutation accumulation lines

Table S8. Summary of 45 confirmed SNPs among the mutation accumulation lines

Table S9. Number of SNP and mutation rate of lotus chromosomes

Table S10. Summary of SNP and Indels in wild lotus accessions

Table S11. 16 models tested in fastsimal2.6

Table S12. Annotation of the selected genes between tropical and temperate lotus

Table S13.GO enrichment analysis of the selected genes between tropical and temperate lotus

Table S14. Annotation of the selected genes between the two subgroups of temperate lotus

Table S15. GO enrichment analysis of the selected genes between the two subgroups of temperate lotus
